# SPOP-mediated ubiquitination and degradation of PDK1 suppresses AKT kinase activity and oncogenic functions

**DOI:** 10.1186/s12943-021-01397-5

**Published:** 2021-08-05

**Authors:** Qiwei Jiang, Nana Zheng, Lang Bu, Xiaomei Zhang, Xiaoling Zhang, Yuanzhong Wu, Yaqing Su, Lei Wang, Xiaomin Zhang, Shancheng Ren, Xiangpeng Dai, Depei Wu, Wei Xie, Wenyi Wei, Yasheng Zhu, Jianping Guo

**Affiliations:** 1 Institute of Precision Medicine, the First Affiliated Hospital, Sun Yat-Sen University, Guangzhou, 510275 Guangdong China; 2 National Clinical Research Center for Hematologic Diseases, Jiangsu Institute of Hematology, The First Affiliated Hospital of Soochow University, Suzhou, China; 3 Key Laboratory of Organ Regeneration and Transplantation of the Ministry of Education, Institute of Immunology, The First Hospital, Jilin University, Jilin Changchun, China; 4 National-Local Joint Engineering Laboratory of Animal Models for Human Diseases, Jilin Changchun, China; 5 State Key Laboratory of Oncology in South China, Collaborative Innovation Center for Cancer Medicine, Sun Yat-Sen University Cancer Center, Guangzhou, 510060 Guangdong China; 6 Department of Urology, Shanghai Changhai Hospital, Shanghai, 200433 China; 7 Department of Pathology, Beth Israel Deaconess Medical Center, Harvard Medical School, Boston, MA 02215 USA

**Keywords:** PDK1, SPOP, AKT, Ubiquitination, Tumorigenesis

## Abstract

**Background:**

3-phosphoinositide-dependent protein kinase-1 (PDK1) acts as a master kinase of protein kinase A, G, and C family (AGC) kinase to predominantly govern cell survival, proliferation, and metabolic homeostasis. Although the regulations to PDK1 downstream substrates such as protein kinase B (AKT) and ribosomal protein S6 kinase beta (S6K) have been well established, the upstream regulators of PDK1, especially its degrader, has not been defined yet.

**Method:**

A clustered regularly interspaced short palindromic repeats (CRISPR)-based E3 ligase screening approach was employed to identify the E3 ubiquitin ligase for degrading PDK1. Western blotting, immunoprecipitation assays and immunofluorescence (IF) staining were performed to detect the interaction or location of PDK1 with speckle-type POZ protein (SPOP). Immunohistochemistry (IHC) staining was used to study the expression of PDK1 and SPOP in prostate cancer tissues. In vivo and in vitro ubiquitination assays were performed to measure the ubiquitination conjugation of PDK1 by SPOP. In vitro kinase assays and mass spectrometry approach were carried out to identify casein kinase 1 (CK1) and glycogen synthase kinase 3 (GSK3)-mediated PDK1 phosphorylation. The biological effects of *PDK1* mutations and correlation with *SPOP* mutations were performed with colony formation, soft agar assays and in vivo xenograft mouse models.

**Results:**

We identified that PDK1 underwent SPOP-mediated ubiquitination and subsequent proteasome-dependent degradation. Specifically, SPOP directly bound PDK1 by the consensus degron in a CK1/GSK3β-mediated phosphorylation dependent manner. Pathologically, prostate cancer patients associated mutations of *SPOP* impaired PDK1 degradation and thus activated the AKT kinase, resulting in tumor malignancies. Meanwhile, mutations that occurred around or within the *PDK1* degron, by either blocking SPOP to bind the degron or inhibiting CK1 or GSK3β-mediated PDK1 phosphorylation, could markedly evade SPOP-mediated PDK1 degradation, and played potently oncogenic roles via activating the AKT kinase.

**Conclusions:**

Our results not only reveal a physiological regulation of PDK1 by E3 ligase SPOP, but also highlight the oncogenic roles of loss-of-function mutations of *SPOP* or gain-of-function mutations of *PDK1* in tumorigenesis through activating the AKT kinase.

**Supplementary Information:**

The online version contains supplementary material available at 10.1186/s12943-021-01397-5.

## Background

Phosphoinositide 3-kinases (PI3K)-AKT signaling pathway plays crucial roles in modulating cell survival, proliferation and metabolic homeostasis, aberrations of which often result in metabolic disorders such as diabetes and cancers [1, 2]. Thus, dysregulations of PI3K-AKT signaling pathway are frequently occurred in various types of tumors (around 50% of human cancers), including but not limited to the amplification/gain-of-function mutations of *PIK3CA*, Ki-ras2 Kirsten rat sarcoma viral oncogene homolog (*KRAS*), *PDPK1* (encoding PDK1) and *AKT*, or deletion/loss-of-function mutations of phosphatase and tensin homolog (*PTEN*), neurofibromatosis type 1 (*NF1*)*,* Von Hippel-Lindau (*VHL*) and protein phosphatase 2A (*PP2A*) [2]. Of note, PDK1 specifically phosphorylates AKT-Thr308 and cooperates with mammalian target of rapamycin complex 2 (mTORC2)-mediated AKT-Ser473 phosphorylation to fully activate the AKT kinase [3–5]. Meanwhile, multiple other post-translational modifications have also been demonstrated to directly dictate AKT kinase activity [6]. Moreover, besides AKT, many other AGC family members including S6K, serum- and glucocorticoid-inducible protein kinase (SGK), polo-like kinase 1 (PLK), ribosomal S6 kinase (RSK) and protein kinase C (PKC), also undergo PDK1-mediated phosphorylation and activation, contributing to diverse biological functions of PDK1 [7–10]. However, the direct regulation of PDK1 is less studied in part due to its auto-phosphorylation and activation property [3, 4]. Recently, accumulating evidence shows that the increased PDK1 protein abundance induced by the genomic amplification robustly contributes to its oncogenic functions in promoting tumorigenesis in different types of cancers [11–13]. Therefore, unraveling the upstream regulation of PDK1 protein turnover will likely provide insights for understanding PI3K-AKT roles in tumorigenesis, and potential targets for combating hyperactive-AKT-driven cancers.

Prostate cancer (PrCa) is the most commonly diagnosed malignancy for men, which leads to the secondary mortality in western countries [14]. Although the castration treatment with androgen receptor (AR) antagonist is an effective therapeutic approach for prostate cancer, many patients become marked resistance to treatment and further progressed to castration-resistant disease (CRPC) [15]. Recently significant efforts have been devoted to exploring potential mechanisms and new targets for CRPC, among which whole genome sequencing studies have successfully illustrated the unanticipated roles of driver mutations in cancers [16]. Of note, around 15% of the Cullin-based ubiquitin ligase adaptor *SPOP* mutations have been identified in PrCa setting [17–19]. Notably, mutant SPOP can dimerize with the wild type (WT) counterpart to repress WT SPOP tumor suppressive functions [20]. In echoing the tumor suppressor roles of SPOP, growing body of evidence show that substrates of SPOP, such as AR [21], steroid receptor coactivator 3 (SRC-3) [22], DEK proto-oncogene (DEK) [23], tripartite motif containing 24 (Trim24) [15], ETS related gene 1 (ERG) [24], bromodomain-containing protein 4 (BRD4) [25] and proto-oncogene c-Myc (c-MYC) [26], play malignant roles in the consequence of *SPOP* loss-of-function mutations in PrCa.

Importantly, the *Spop* conditional prostate knockout mice have been generated and display dramatically increased AKT kinase activity [27]. Meanwhile, another study focused on SPOP-mediated BRDs degradation also revealed that depleted *SPOP* could induce AKT kinase activity potentially by upregulating BRDs downstream genes [28]. In contrast, SPOP has been considered as an oncogene in kidney cancer or cancers under hypoxic conditions to promote AKT kinase activity by degrading the tumor suppressor PTEN [29]. These findings collectively indicate that SPOP plays distinct roles in modulating AKT activity in a tissue context-dependent manner. In this study, we identify that Cullin3^SPOP^ E3 ligase promotes PDK1 ubiquitination and subsequent degradation via a CRISPR-based enzymatic screening approach. Mechanistically, SPOP recognizes PDK1 in a CK1/GSK3β-mediated phosphorylation and degron dependent manner. Either loss-of-function mutations of *SPOP* or gain-of-function mutations of *PDK1* in their binding region all attenuate SPOP recognizing and ubiquitinating PDK1, leading to elevat PDK1 protein abundance, AKT kinase activity and benefit of tumor malignancies. Thus, these findings reveal a fine-tune regulation of PDK1 turnover by SPOP-mediated ubiquitination, and highlight the PDK1-AKT pathway will be a potential target for mutated *SPOP-* or *PDK1*-driven cancers.

## Methods

### Cell culture, transfection and cell fractionations

Human embryonic kidney 293 (HEK293), HEK293T and DLD1 cells were cultured in DMEM with 10% fetal bovine serum (FBS), 100 units of *penicillin* and 100 μg/ml *streptomycin*. Prostate cancer cell line C4-2, 22Rv1, PC3 and DU145 were maintained in RPMI 1640 medium supplemented with 10% FBS. *Spop*^+*/*+^ and *Spop*^*−/−*^ mouse embryonic fibroblasts (MEFs) were a gift from N. Mitsides (Baylor College of Medicine). DLD1-*PDK1*^*−/−*^ and counterpart cells were kindly provided by Dr. Bert Vogelstein (Johns Hopkins University School of Medicine), and these cells were also maintained in DMEM medium supplemented with 10% FBS. Cell transfection was carried out as described previously [30]. Lentiviral small hairpin RNA (shRNA) virus packaging and infection were performed according to the previous protocol [31]. CK1 inhibitor IC261 (Calbiochem, 400090), D4476 (Sigma, D1994) and GSK inhibitor CHIR-99021 (Selleck, S2924) were used at the dose as indicated. Cycloheximide (CHX) assays were executed as described previously [32].

### Plasmid construction

Myc-tagged Cullin1, Cullin2, Cullin3, Cullin4A, Cullin4B and Cullin5, CK1α, CK1ε, CK1δ, CK1γ were described previously [24]. Flag-PDK1, HA-PDK1, Myc-PDK1, pLenti-PDK1, pET-28a-His-PDK1, pGEX-4T-1-PDK1, CMV-GST-PDK1, CMV-GST-PDK1-N, CMV-GST-PDK1-KD, CMV-GST-PDK1-PolyS, CMV-GST-PDK1-PH were constructed by sub-cloning the corresponding cDNAs into pCDNA3, pLenti-puro, pET-28a, pGEX-4T-1 and pCMV-GST, respectively. Various PDK1 mutants were generated via QuikChange XL Site-Directed Mutagenesis Kit (Stratagene) according to the manufacturer’s instructions. SPOP-related constructs were generated as described previously [24]. Flag-Keap1 was purchased from Addgene. Flag-COP1 and Flag-DET1 constructs were kind gift from Dr. William Kaelin (Dana-Farber Cancer Institute). Details of plasmid constructions are provided upon request.

### Antibodies

All antibodies were used at a 1:1000 dilution in 5% non-fat milk for western blot. Anti-PDK1 antibody (8292787) were purchased from BD Biosciences. Anti-SPOP antibody (16750–1-AP) and anti-CK1 antibody (14388–1-AP) were purchased from Proteintech. Anti-pS473-AKT antibody (4060), anti-pT308-AKT antibody (2965), anti-AKT1 antibody (2938), anti-AKT total antibody (4691), anti-Cullin 3 (2759), anti-GST (2625), polyclonal anti-Myc-Tag antibody (2278), anti-GAPDH (5174) and monoclonal anti-Myc-Tag (2276) antibodies were purchased from Cell Signaling Technology. Monoclonal anti-HA antibody (MMS-101P) was purchased from Biolegend. Anti-TRIM24 (C-4), polyclonal anti-HA (SC-805) and anti-p27 (SC-528) were purchased from Santa Cruz. Polyclonal anti-Flag antibody (F-2425), monoclonal anti-Flag antibody (F-3165, clone M2), anti-Tubulin antibody (T-5168), anti-Vinculin antibody (V-4505), anti-Flag agarose beads (A-2220), anti-HA agarose beads (A-2095), peroxidase-conjugated anti-mouse secondary antibody (A-4416) and peroxidase-conjugated anti-rabbit secondary antibody (A-4914) were purchased from Sigma.

### CRISPR/Cas9 library screening

A lentiviral reporter vector plenti-hygro-CMV-DsRed-IRES-EGFP-PDK1 which encoded DsRed and green fluorescent protein (EGFP)-PDK1 proteins respectively was generated. The reporter system was stably integrated into HEK293T cells and selected with hygromycin to stable clones. Then, the reporter cells were infected with lentivirus carrying the E3 ubiquitin ligase contained CRISPR-Cas9 library, and selected for 7 days with the treatment of puromycin. The CRISPR-Cas9 library-containing reporter cells were subjected to flow cytometry sorting, and cells with drastically either enhanced or decreased fluorescence intensities of EGFP/DsRed were collected for genomic deoxyribonucleic acid (DNA) extraction. And the CRISPR library single guide RNAs (sgRNAs) were amplified by polymerase chain reaction (PCR) and then high throughput sequenced. After alignment, the differentially expressed sgRNAs were calculated and the corresponding genes are ranked.

### shRNAs, sgRNAs and CRISPR/Cas9-mediated knockout assay

shRNA vectors to deplete endogenous *SPOP* were obtained from Sigma (TRCN0000122224, TRCN0000139181, TRCN0000145024). Knockout *PDK1* with CRISPR/Cas9 system has been performed as we have done previously [33] with sgRNA: sg*PDK1.* Forward: 5’-CACCGcaagtttgggaaaatccttg-3; Reverse: 5’-AAACcaaggattttcccaaacttgC-3’. *SPOP* knockout cell lines were generated as previously reported [25].

### Immunoprecipitation (IP), GST pull-down (PD) assays and western blot

Cells were lysed in EBC buffer (50 mM Tris pH 7.5, 120 mM NaCl, 0.5% NP-40) with phosphatase inhibitors (phosphatase inhibitor cocktail set I and II, Calbiochem) and protease inhibitors (Complete Mini, Roche). The protein concentrations of lysates were measured by the Beckman Coulter DU-800 spectrophotometer using the Bio-Rad protein assay reagent. Same amounts of whole cell lysates (WCL) were resolved by sodium dodecyl sulfate polyacrylamide gel electrophoresis (SDS-PAGE) and immunoblotted with indicated antibodies. For immunoprecipitation, 1000 μg lysates were incubated with the indicated antibody (1–2 μg) for 3–4 h at 4 °C followed by 1 h incubation with Protein A sepharose beads (GE Healthcare). Immunoprecipitants were washed five times with NETN buffer (20 mM Tris, pH 8.0, 100 mM NaCl, 1 mM EDTA and 0.5% NP-40) before being resolved by SDS-PAGE and immunoblotted with indicated antibodies. Quantification of the immunoblot band intensity was performed with ImageJ software.

### In cell ubiquitination assays

In cell ubiquitination assays were performed as previously described [32]. Briefly, HEK293T cells were transfected with His-Ub and the indicated constructs. 36 h post-transfection, resulting cells were treated with 10 μM carbobenzoxy-Leu-Leu-leucinal (MG132) for 12 h and washed with PBS twice, and then were lysed in buffer A (6 M guanidine-HCl, 0.1 M Na_2_HPO_4_/NaH_2_PO_4_, and 10 mM imidazole (pH 8.0)) and subjected to sonicate. After high-speed centrifuged, the supernatants were incubated with nickel-beads (Ni–NTA) (Qiagen) for 3 h at room temperature. The products were washed twice with buffer A, twice with buffer A/TI (1 volume buffer A and 3 volumes buffer TI), and one time with buffer TI (25 mM Tris–HCl and 20 mM imidazole (pH 6. 8)). The pull-down proteins were resolved in 8% SDS-PAGE for immunoblot analysis.

### In vitro ubiquitination assays

In vitro ubiquitination assays were performed as described previously [28]. Briefly, HEK293T cells were transfected with HA-SPOP (WT, Y87F, F102C, Y183F) to purify various SPOP by HA affinity precipitation. 1 μg of bacterially purified His-PDK1 was incubated with purified SPOP together with E1, E2 (UbcH5a and UbcH3) and ubiquitin (obtained from UBbiotech) in the reaction buffer. The reaction was performed at 37 °C for 2 h and stopped by 2xSDS sample buffer, then resolved by SDS-PAGE for immunoblotting.

### In vitro* kinase* assays

PDK1 in vitro kinase assays were adapted from a protocol described previously [32]. Briefly, 1 μg of the bacterially purified His-fusion PDK1 protein were incubated with immunoprecipitated CK1 from cell lysates in the presence of 200 μM adenosine triphosphate (ATP) (with ^32^P-ATP) in the kinase reaction buffer (50 mM Tris-HCl pH 7.5, 1 mM MnCl_2_, 2 mM DTT, 1 mM EGTA) for 30 min at 30 °C. The reaction was subsequently stopped by the addition of 3xSDS loading buffer and resolved by SDS-PAGE. Phosphorylation of his-PDK1 was detected by autoradiography.

### Purification of GST- and His-tagged proteins from bacteria

Recombinant GST-conjugated PDK1 was generated by transforming the BL21 (DE3) *E. coli* strain with pGEX-4T1-PDK1 or pGEX-4T1-1 (Empty vector control). Starter cultures grown overnight at 37 °C were inoculated (1%) into larger volumes. Cultures were grown at 37 °C until an O.D. 0.8, following which protein expression was induced for 16 h using 0.1 mM isopropyl β-d-1-thiogalactopyranoside (IPTG) at 16 °C. Pellets were re-suspended in EBC buffer and sonicated. The supernatant was incubated with Glutathione-sepharose slurry (GE) for 2 h at 4 °C. The Glutathione beads were washed 3 times with PBS buffer and stored at 4 °C in EBC buffer or eluted by elution buffer and further analyzed by Coomassie blue staining and quantified by bovine serum albumin (BSA) standards.

The recombinant His-PDK1 was generated by transforming the BL21 (DE3) *E. coli* strain with by the same strategy as GST-tagged proteins. The difference was that the supernatant was incubated with Nickel resin slurry (Qiagen) for 2 h at 4 °C. The Nickel resins were washed 4 times with Tris-buffered saline (TBS) buffer (50 mM Tris-HCl pH 8.0, 120 mM NaCl) containing 10 mM imidazole (Sigma) and eluted by TBS buffer containing 100 mM imidazole.

### Peptide synthesis and pulldown assay

Hypoxia-inducible factor 1 alpha (HIF1α) and PDK1 associated peptides used for dot blot assays were synthesized by Sangon Biotechnology. The sequences were listed as below:

- HIF1: Bio-RLQFDDDMPIYPALMELDLD
- PDK1-WT: Bio-FGCMQVSSSSSSHSLSA
- PDK1-Δ6S: Bio-FGCMQVHSLSA
- PDK1-Q387R: Bio-FGCMRVSSSSSSHSLSA
- PDK1-Q387H: Bio-FGCMHVSSSSSSHSLSA
- PDK1-S390L: Bio-FGCMQVSLSSSSHSLSA

Peptides were diluted into 2 mg/ml for further pulldown assays: 4 μg peptides were incubated and rocked with streptavidin beads for 1 h at 4 °C, and the beads were washed twice with NETN buffer. The peptides conjugated beads were incubated with cell lysis transfected with SPOP for another 4 h, and then the pulldown products were washed four times with NETN buffer and subjected for immunoblot analysis.

### IHC staining

The treatment-naive prostate tumor specimens were obtained from Shanghai Changhai Hospital in China and usage of these specimens was approved by the Institute Review Board of Shanghai Changhai Hospital. Informed consent was obtained from each patient. The *SPOP* mutation status was determined as has been previously reported [28, 34]. Paraformaldehyde fixed paraffin embedded prostate cancer samples were deparaffinized, rehydrated, and subjected to heat-mediated antigen retrieval. The UltraSensitive TM SP (rabbit) IHC Kit (KIT-9706, Fuzhou Maixin Biotech) was used by following the manufacturer’s instructions with minor modification. Briefly, the sections were incubated with 3% H_2_O_2_ for 15 min at room temperature to eliminate the endogenous peroxidase activity. After incubating in normal goat serum for 1 h, sections were treated with primary PDK1 antibody (dilution 1:300; Abcam; ab52893) at 4 °C overnight. The sections were then washed 3 times in 1 x phosphate-buffered saline (PBS) and treated with biotinylated goat-anti-rabbit IgG secondary antibodies for 30 min followed by incubating with streptavidin-conjugated HRP for 15 min. After washing three times in 1xPBS for 5 min each, specific detection was developed with 3′3-diaminobenzidine (DAB-2031, Fuzhou Maixin Biotech). Images were taken by an Olympus camera and matched software. The expression level of PDK1 in prostate cancer samples was scored according to the intensity of the IHC staining as 1, weak expression; 2, intermediate expression and 3, strong expression. The Mann–Whitney test for independent samples was used to compare the difference of PDK1 expression between *SPOP* mutated and wild type cases. *p* < 0.05 was considered as significant.

### Mass spectrometry analyses

For mass spectrometry (MS) analysis, anti-Flag IPs were performed with the WCL derived from three 10 cm dishes of HEK293T cells transfected with Flag-PDK1 with/without CK1 or GSK3β and treated with MG132. The proteins were resolved by SDS-PAGE, and identified by Coomassie staining. The band containing PDK1 was reduced with 10 mM Dithiothreitol (DTT) for 30 min, alkylated with 55 mM iodoacetamide for 45 min, and in-gel-digested with trypsin enzymes. The resulting peptides were extracted from the gel and analyzed by microcapillary reversed-phase (C_18_) liquid chromatography-tandem mass spectrometry (LC–MS/MS), using a high resolution QExactive HF Orbitrap (Thermo Fisher Scientific) in positive ion DDA mode (Top 8) via higher energy collisional dissociation (HCD) coupled to a Proxeon EASY-nLc II nano- high performance liquid chromatography (HPLC). MS/MS data were searched against the Uniprot Human protein database (version 20151209 containing 21,024 entries) using Mascot 2.5.1 (Matrix Science) and data analysis was performed using the Scaffold 4.4.8 software (Proteome Software). Peptides and modified peptides were accepted if they passed a 1% false discovery rate (FDR) threshold.

### Colony formation assays

Cells were seeded into 6-well plates in medium (300 or 600 cells/well) and cultured for two weeks until the colonies are visible. Colonies were washed with PBS and fixed by 10% acetic acid/10% methanol for 20 min, then the colonies were stained with 0.4% crystal violet in 20% ethanol for 20 min. Then the plates were washed by H_2_O and air-dried, and colonies were numbered. Three independent experiments were performed to generate the SD.

### Soft agar assays

The assays were preformed using 6-well plates where the solid medium consists of two layers. 2% melting Nobel agar was prepared and mixed with RPMI 1640 to make the 0.4% and 0.8% agar in 50 °C. 0.8% agar were added to the bottom layer and the 0.4% agar suspended with 1 × 10^4^ or 3 × 10^4^ cells were added to the top layer. Then add 500 μl complete RPMI 1640 medium to keep the top layer moisture. After 4–6 weeks, the cells were stained with iodonitrotetrazolium chloride (1 μg/ml) (Sigma, I10406) for colony visualization and counting. Three independent experiments were performed to generate the standard deviation (SD).

### Cell proliferation assays

The cells were seeded in the 96-well plates (5000 cells/well). At the indicated time points, the cell proliferation was detected with MTS assay (Promega, G3530) according to the manufacturer’s instructions.

### Mouse xenograft assays

Mouse xenograft assays were performed as described previously [33]. Briefly, 5 × 10^6^ DLD1-*PDK*^*−/−*^ cells stably expressing WT or mutant forms of PDK1 mixed with matri-gel were injected into the flank of 8 female nude mice (University of Sun Yat-sen, 4–5 weeks of age). Tumor size was measured every two days with a caliper, and the tumor volume was determined with the formula: L x W^2^ × 0.5, where L is the longest diameter and W is the shortest diameter.

### Quantification and statistical analysis

Some key experiments have been repeated 3 times, quantitated, and subjected to statistical analysis. Statistical associations between experimental groups was tested by Student’s *t*-test, or one-way analysis of variance (ANOVA) test using the Prism 7 Software. The difference of PDK1 expression in prostate cancers was analyzed with Chi-squared (χ^2^) test. Simultaneous 95% confidence bands were computed for the whole range of time values. The threshold for statistical significance was set to *p* < 0.05.

## Results

### CRISPR-based screening of PDK1 E3 ligase

**Fig. 1:**
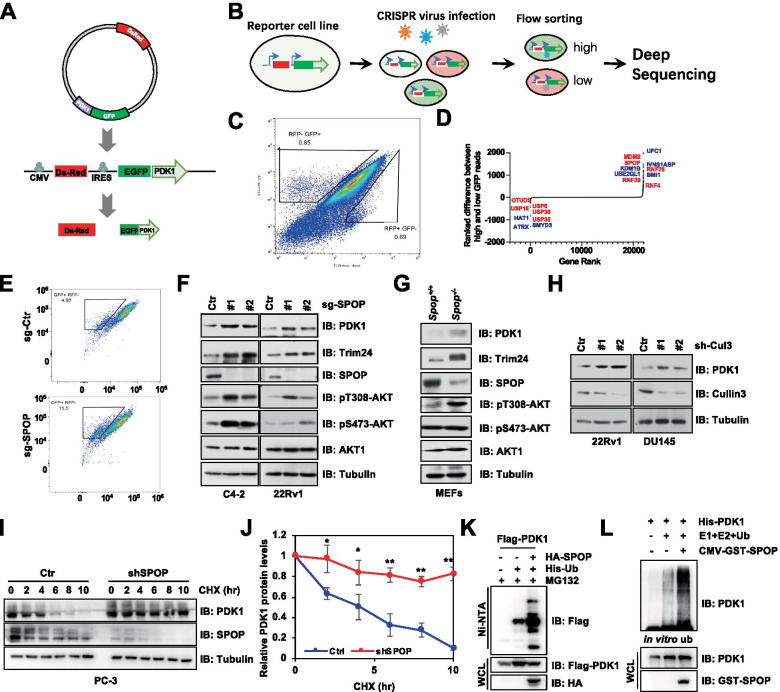
CRISPR screen identifies SPOP as a *bona fide* E3 ligase for PDK1. **A** A schematic illustration for the plenti-hygro-CMV-DsRed-IRES-EGFP-PDK1 reporter system. **B** Flow-chart of genome-scale library screening using CRISPR/Cas9 system. **C** The selected reporter cells were sorted by flow cytometry. **D** Identification of top candidate sgRNA genes using a scatter plot after high-throughput sequencing. **E** Stable expression of DsRed/EGFP-PDK1 HEK293T cells were infected with lenti-CRISPR sgRNAs targeting control or *SPOP* for 7 days and then analyzed by flow cytometry. **F** Immunoblot (IB) analysis of WCL derived from C4-2 and 22Rv1 cells. Where indicated endogenous *SPOP* was knockout by using CRISPR/Cas9. **G** IB analysis of WCL derived from *Spop*^*−/−*^ and counterpart MEFs. **H** IB analysis of WCL derived from RV1 and DU145 cells transfected with indicated lentiviral shRNAs. The transfected cells have been selected with puromycin (1 μg/ml) for 72 h to eliminate the uninfected cells before harvested. **I**, **J** IB analysis of control and *SPOP* knockdown PC3 cells. Where indicated cells were treated with CHX (100 μg/ml) for the indicated time points before harvested. PDK1 protein abundance in (**I**) was normalized and quantified (H) (mean ± SD, n = 3) (*t* test), *P < 0.05, **P < 0.01. **K** IB analysis of WCL and His pulldown products derived from HEK293T cells transfected with indicated constructs. Cells were treated with MG132 (10 μM) for 12 h before harvested. **L** In vitro ubiquitination assays were performed with commercial E1, E2 and Ub proteins, bacterially purified His-PDK1, as well as SPOP purified from SPOP-overexpressing HEK293T cells. The reaction products were subjected to IB analysis

Although PDK1 has been demonstrated as an “un-regulable” kinase because of the auto-phosphorylation and activation mechanism [3, 4], the genetic amplification of *PDK1* in diverse cancers has been considered to promote PDK1 oncogenic functions [13]. Therefore, the potential E3 ligase which targets PDK1 for degradation will likely play an important role in regulating PDK1 oncogenic functions. To this end, we initially observed that the protein levels of PDK1 were significantly elevated following the treatment of proteasome inhibitor MG132 in different cell lines (Fig. S1A), indicating that PDK1 protein is subjected to the proteasomal degradation system. After that, we applied a well-established CRISPR-based screening approach to identify the potential upstream E3 ubiquitin ligase for PDK1 [35]. Briefly, an EGFP-tagged PDK1 fusion expression reporter cell line that monitored PDK1 protein stability was generated with DsRed expression as a control in HEK293T cells (Fig. 1A). Validating the ability of this reporter to reliably read-out the PDK1 steady was performed by treatment with MG132 (Fig. S1B). Furthermore, an E3 ubiquitin ligase and deubiquitinase contained CRISPR-Cas9 screening system was performed by infecting EGFP-PDK1 reporter cells with a lentiviral CRISPR library (Fig. 1B, S1C) [35]. In the end, the populations at the top 0.85% and the bottom 0.69% of GFP/RFP ratio, expected to be enriched and depleted PDK1 expression respectively, were collected and subjected for further deep sequencing (Fig. 1C).

Among the higher versus lower EGFP expression hits, several E3 ligases, such as mouse double minute 2 homolog (MDM2), SPOP, ring finger protein 26 (RNF26) and RNF29, were ranked in the top to enhance EGFP-PDK1 signals in our screening system (Fig. 1D). Furthermore, we chose these sgRNAs targeting different E3 ligases to detect the EGFP-PDK1 expression, and observed that the sgRNA targeting *SPOP* could markedly increase EGFP-PDK1 level detected with either western blot or flowcytometry for the ratio of EGFP/DsRed (Fig. 1E, S1D-E). Importantly, we further observed that depletion of *SPOP* with different sgRNAs could significantly elevate endogenous PDK1 protein levels, as well as well-established SPOP substrate Trim24 in prostate cancer cell lines (Fig. 1F). Similar result was also observed in the *Spop* knockout MEFs (Fig. 1G). Collectively, our results conclude that SPOP is a potential PDK1 E3 ligase.

### SPOP directly interacts with and ubiquitinates PDK1

As SPOP contains a Bric-a-brac (BTB) domain and belongs to Cullin3-based E3 ligase family [36], the largest class of E3 ubiquitin ligases ever found [37], we initially examined the connection of PDK1 with the Cullin-family proteins. We found that Cullin3 but not other Cullin proteins specifically interacted with PDK1 (Fig. S1F). In support of this notion, depletion of endogenous *Cullin3* led to an evident increase of endogenous PDK1 in prostate cancer 22Rv1 and DU145 cells (Fig. 1H), indicating that Cullin3-based E3 ligase could govern PDK1 protein stability. Next, we investigated the potential binding of PDK1 with a panel of Cullin-binding E3 ligases, and observed that PDK1 interacted with SPOP (Fig. S1G-H), but not with COP1/DET1 and KEAP1, another Cullin 3/4-related E3 ligases in cells. Consistent with this finding, SPOP, but not COP1 or KEAP1 could dramatically decrease PDK1 protein levels (Fig. S1I), and promoted PDK1 protein degradation in a dose-dependent manner (Fig. S1J-K). Furthermore, we detected the half-life of PDK1 in *SPOP* depleted PC3 and C4-2 cells, and observed that PDK1 protein stability was prolonged in *SPOP*-deficient cells compared with parental cells (Fig. 1I-J, S1L). In line with these findings, SPOP could markedly promote PDK1 ubiqutination both in cells and in vitro (Fig. 1K-L). Taken together, PDK1 is a *bona fide* substrate of the Cullin3^SPOP^ E3 ligase complex.

In keeping with another two reports [27, 28], we analyzed the database and observed that *SPOP* loss-of-function mutations were mutually exclusive with *PTEN* deletion/loss-of-mutations (Fig. S1M), indicating that SPOP might tightly relate to AKT activation. Moreover, both in *SPOP* knockout prostate cancer cells and MEFs, *SPOP* depletion could enhance PDK1 protein levels coupled with increased AKT phosphorylation in T308 (Fig. 1F-G). However, it has been previously reported that SPOP could upregulate AKT phosphorylation by directly degrading PTEN when it resided in the cytoplasm under hypoxic conditions [29]. To explain these controversial findings, we sought to investigate whether nuclear, but not cytoplasm, SPOP could degrade PDK1 protein. To this end, we generated a cytoplasm specifically localized SPOP-Δ366 (also termed cSPOP) [29], and observed that compared with WT-SPOP, cSPOP diminished its binding with and ubiquitination of PDK1 (Fig. S2A-B), resulting in mildly affecting PDK1 abundance (Fig. S2C-D) and half-life span (Fig. S2E-F). To further refine the distinct regulation of SPOP on AKT signaling in a location dependent manner, we separated the cell fraction and observed that cytoplasm localized cSPOP could promote PDK1 membrane translocation, possibly due to suppressing PTEN expression and PIP_3_ generation (Fig. S2G) [29]. In keeping with this finding, we performed IF staining and observed that PDK1 partially co-localized with SPOP in nucleus, but less co-localized with cSPOP in the cytoplasm (Fig. S2H). In addition, we also found that PDK1 was prone to reside in cytoplasm membrane upon cSPOP expression. Taken together, our data indicate that in different tissues or cellular compartmental conditions, SPOP might play different roles as tumor suppressor or oncogene in manipulating AKT kinase by degrading distinct substrates such as PDK1 or PTEN, respectively (Fig. S2I).

### Prostate cancer patients associated *SPOP* mutants fail to interact with and degrade PDK1

**Fig. 2:**
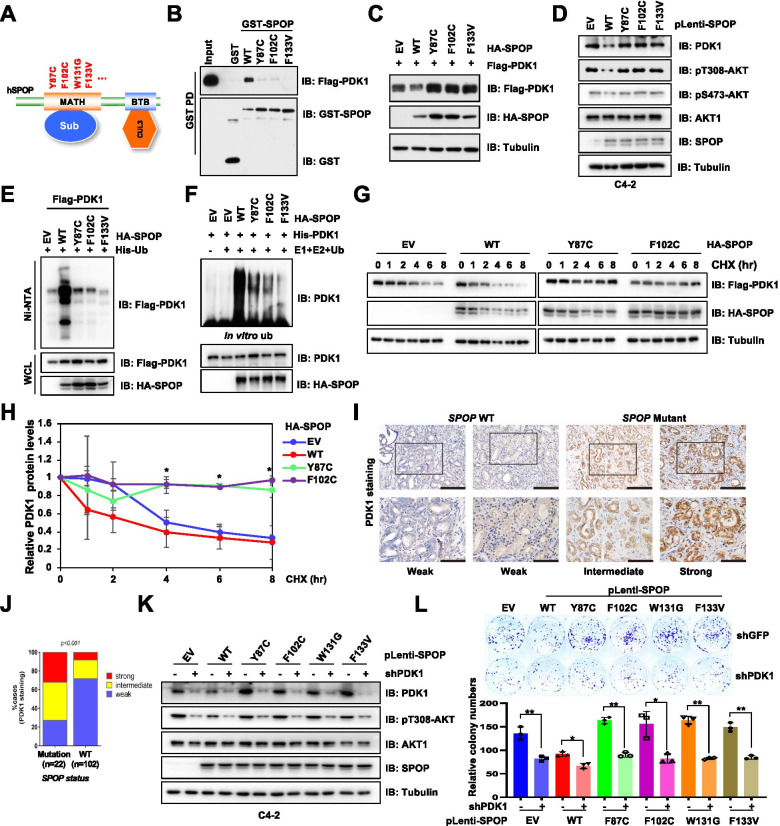
Patient-associated mutant forms of *SPOP* dictate PDK1 stability and oncogenic function. **A** A schematic illustration of SPOP domains and frequently occurred prostate cancer-associated mutations. **B** IB analysis of WCL and GST pulldown products derived from HEK293T cells transfected with Flag-PDK1 and GST-SPOP (WT, Y87C, F102C, F133V). Cells were treated with MG132 (10 μM) for 10 h before harvested. **C** IB analysis of WCL derived from HEK293T cells transfected with indicated constructs. **D** IB analysis of WCL derived from C4-2 cells stably expressing SPOP WT or mutants. **E** IB analysis of WCL and His pulldown products derived from HEK293T cells transfected with indicated constructs. Cells were treated with MG132 (10 μM) for 12 h before harvesting. **F** In vitro ubiquitination assays were performed with commercial E1, E2 and Ub proteins, bacterially purified His-PDK1, as well as SPOP purified from SPOP-overexpressing HEK293T cells. The reaction products were subjected to IB analysis. **G**, **H** IB analysis WCL derived from of HEK293T cells transfected with indicated constructs. Where indicated cells were treated with CHX (100 μg/ml) for the indicated time points before harvested. PDK1 protein abundance in (**G**) was normalized and quantified (**H**) (mean ± SD, n = 3) (*t* test), *P < 0.05. **I**-**J** IHC staining was performed in prostate cancer tissues, where indicated, WT *SPOP* tissues displayed weak PDK1 staining, whereas, mutant *SPOP* tissues displayed intermediate and strong PDK1 staining (**I**). Scale, top: 100 μm; bottom: 200 μm. The expression of PDK1 was calculated depending on *SPOP* genetic status and quantified in (**J**). Chi-squared (χ^2^) test, *P* < 0.001. (K-L) C4-2 cell lines stably expressed prostate cancer associated *SPOP* mutants were infected with shGFP or shPDK1 lentivirus. Cells were selected with puromycin (1 μg/ml) for 72 h to eliminate uninfected cells and used for IB analyses. Resulting cells were subjected to colony formation assays (L, top panel), quantified and plotted (L, bottom panel) (mean ± SD, n = 3) (*t* test), *P < 0.05, **P < 0.01

Given that SPOP could recognize substrates via its Meprin and TRAF homology (MATH) domain [38], we found that deletion of MATH domain could markedly abolish SPOP interaction with PDK1 (Fig. S3A). In support of this notion, deletion of either MATH or BTB domain which is important for SPOP binding with substrate and Cullin3 respectively could abrogate SPOP-induced degradation of PDK1 (Fig. S3B). Recently, the genome sequencing studies have disclosed that *SPOP* is one of the most frequently mutated genes (up to 15% of cases) in prostate cancers [17, 39], among which most of *SPOP* somatic mutations occurred within its MATH domain (Fig. 2A). In accordance with the previous finding that *SPOP* mutants impaired its ability to recognize substrates [38, 40], we observed that these *SPOP* mutants failed to interact with PDK1 (Fig. 2B) and degrade PDK1, c-MYC and PTEN (Fig. 2C-D, S3C). Similar result was also observed in cSPOP mutants, indicating that these MATH mutations could block SPOP recognizing substrates (Fig. S3C). Consistently, these *SPOP* mutants also abrogated PDK1 ubiquitination both in cells and in vitro (Fig. 2E-F). Meanwhile, ectopic expression of wild-type *SPOP* (WT-SPOP), but not *SPOP* mutants, led to significant decrease of PDK1 half-life (Fig. 2G-H). In echoing this finding, we detected PDK1 expression in prostate cancer patients, and observed that samples of *SPOP* mutants displayed a relative higher PDK1 expression compared with that of samples harboring intact *SPOP* (Fig. 2I-J). While, several samples bearing *SPOP* mutant still displayed a relatively weak expression of PDK1, possibly due to non-loss-of-function mutations occurred in *SPOP*. Functionally, since prostate cancer cells expressing *SPOP* mutants displayed enhanced colony-formation ability in monolayer culture compared with that of cells expressing intact *SPOP* (Fig. S3D), we observed that *SPOP* mutants-induced colony formation could be largely restricted by depleting *PDK1* (Fig. 2K-L, S3E-F). Therefore, this finding suggests that SPOP exhibits tumor suppressor roles in part via degrading PDK1 in prostate cancer setting.

### SPOP ubiquitinates and degrades PDK1 in a degron dependent manner

**Fig. 3:**
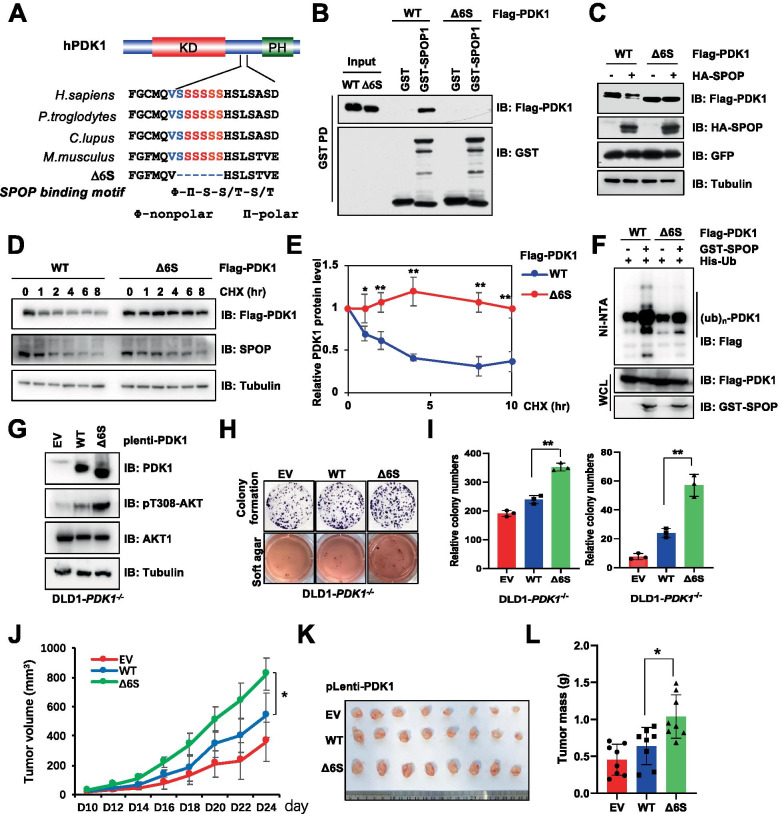
SPOP ubiquitinates and subsequently degrades PDK1 in a degron dependent manner. **A** A schematic presentation of the evolutionarily conserved putative SPOP binding motif in PDK1. **B** IB analysis of WCL and IP products derived from HEK293T cells transfected with indicated constructs. Cells were treated with MG132 (10 μM) for 10 h before harvested. **C** IB analysis of WCL derived from HEK293T cells transfected with Flag-PDK1 (WT, Δ6S) and with/without HA-SPOP. **D-E** IB analysis WCL derived from HEK293T cells transfected with Flag-PDK1 (WT, Δ6S). Where indicated cells were treated with CHX (100 μg/ml) for the indicated time points before harvested. PDK1 protein abundance in (**D**) was normalized and quantified (**E**) (mean ± SD, n = 3) (*t* test), *P < 0.05, **P < 0.01. **F** IB analysis of WCL and His pulldown products derived from HEK293T cells transfected with indicated constructs. Cells were treated with MG132 (10 μM) for 12 h before harvested. **G-I** IB analysis of DLD1-*PDK1* knockout cells stably infected with indicated constructs (**G**). Resulting cells were subjected to colony formation and soft agar assays (**H**), and the relative colony numbers were quantified (I) (mean ± SD, n = 3) (*t* test), **P < 0.01. **J-L** Cells generated in (**G**) were subjected to mouse xenograft assays. Tumor size was monitored (**J**), and dissected tumors were weighed (**K, L**). (mean ± SD, n = 8) (ANOVA test for J, and *t* test for L), *P < 0.05

To point out whether SPOP degrades PDK1 in a degron-dependent manner, we firstly truncated PDK1 protein and observed that SPOP robustly bound PDK1 in its poly serine (PolyS) region (Fig. S3G-H). As a well-studied E3 ligase, SPOP commonly recognizes its substrate in a canonical degron dependent manner with a consensus motif Φ-Π-S–S/T-S/T (Φ, nonpolar; Π, polar) [38]. As such, we scanned the PolyS region of PDK1 and identified one evolutionarily conserved putative SPOP-binding motif (or degron) “VSSSSSS” (Fig. 3A). Furthermore, deletion of this degron (termed as Δ6S) not only disturbed SPOP interacting with PDK1 (Fig. 3B, S3I-J), but also compromised SPOP-mediated PDK1 degradation (Fig. 3C). Consistently, loss of degron could prolong PDK1 half-life compared with that of WT PDK1 (Fig. 3D-E). In support of the role of degron in SPOP-mediated PDK1 ubiquitination, ubiquitination conjugation of PDK1-Δ6S was markedly reduced compared with that of WT-PDK1 (Fig. 3F). Functionally, degron-deleted PDK1 exhibited more oncogenic functions in promoting cell colony formation and anchorage growth compared with that of WT-PDK1 expressed DLD1-*PDK1*^*−/−*^ and HEK293T-sg*PDK1* cells (Fig. 3G-I, S3K), concomitant with an increase of drug resistance to cisplatin and etoposide (Fig. S3L-M). Importantly, non-SPOP recognized PDK1 also promoted tumor growth in mouse, coupled with increased AKT kinase activity (Fig. 3J-L, S3N-O). Collectively, these data underscore that SPOP recognizes and degrades PDK1 in a degron dependent manner.

### Both GSK3β and CK1 phosphorylate PDK1 and lead to PDK1 degradation in a SPOP dependent manner

**Fig. 4:**
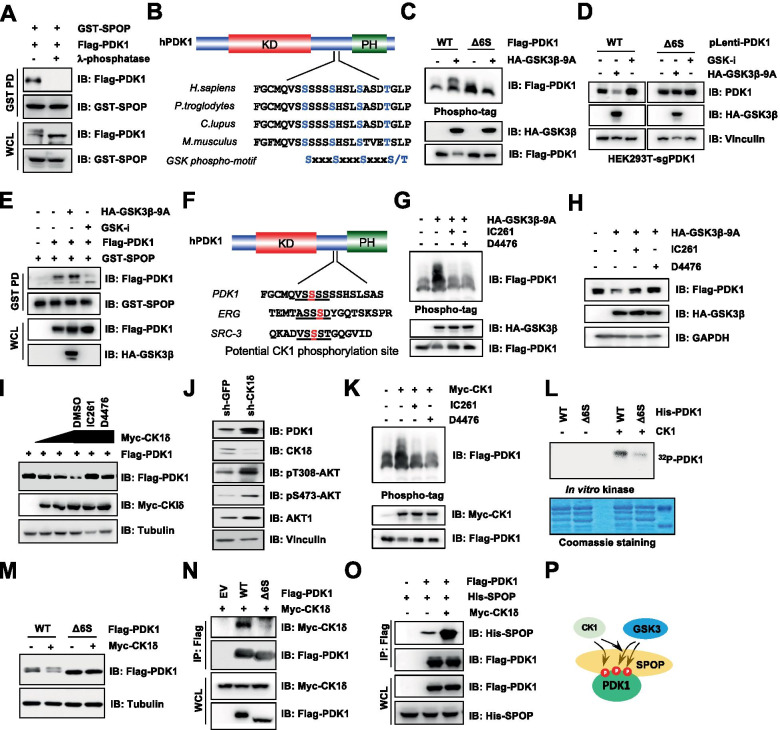
CK1/GSK3-mediated PDK1 phosphorylation promotes SPOP interaction with and ubiquitination of PDK1. **A** IB analysis of WCL and GST-pulldown products derived from HEK293T cells transfected with GST-SPOP, Flag-PDK1 and treated with/without λ-phosphatase. **B** A schematic presentation of the evolutionarily conserved putative GSK phosphorylation motif in PDK1. Red label indicates the potential GSK3 phosphorylation sites. **C** HEK293T cells transfected with indicated constructs were resolved by phospho-tag SDS-PAGE and immunoblotted with indicated antibodies. **D** IB analysis of HEK293T-*PDK1* knockout cells stably infected with indicated constructs. Where indicated cells were treated with GSK3β inhibitor CHIR-99021 (10 μM) for 10 h before harvested. **E** IB analysis of WCL and GST-pulldown products derived from HEK293T cells transfected with indicated constructs. Where indicated, cells were treated with GSK3β inhibitor CHIR-99021 (10 μM) for 10 h before harvested. **F** A schematic presentation of CK1 phosphorylation motif in PDK1 and other well-established SPOP substrates, such as ERG and SRC-3. Red label indicates the potential CK1 phosphorylation site. **G-H** HEK293T cell transfected with indicated constructs were resolved by phospho-tag SDS-PAGE (**G**) or normal SDS-PAGE (**H**), and immunoblotted with indicated antibodies. Where indicated, cells were treated with CK1 inhibitor IC261 (50 μM) or D4476 (20 μM) for 10 h before harvested. **I** IB analysis of WCL derived from HEK293T cells transfected with Flag-PDK1 and increasing Myc-CK1δ. Where indicated, cells were treated with CKI inhibitor IC261 (50 μM) or D4476 (20 μM) for 10 h before harvested. **J** IB analysis of WCL from MCF7 cells transfected with indicated lentiviral shRNA. The transfected cells have been selected in puromycin (1 μg/ml) for 72 h to eliminate the uninfected cells before harvested. **K** HEK293T cells transfected with indicated constructs were resolved by phospho-tag SDS-PAGE and immunoblotted with indicated antibodies. Where indicated, cells were treated with CK1 inhibitor IC261 (50 μM) or D4476 (20 μM) for 10 h before harvested. **L** In vitro kinase assay was performed with bacterially purified PDK1 as substrate, and recombinant CK1 protein as the kinase source ^32^P isotope-ATP was used for autoradiography of phosphorylated PDK1. **M** IB analysis of WCL derived from HEK293T cells transfected with Flag-PDK1 (WT, Δ6S) and with/without Myc-CK1δ. **N–O** IB analysis of WCL and IP products derived from HEK293T cells transfected with Myc-CK1δ and indicated constructs. **P** A schematic model illustrates that CK1 phosphorylating PDK1 could mediate GSK3 phosphorylating PDK1 and in turn promote SPOP recognizing PDK1

Since proper phosphorylation of substrate is commonly desired for the recognition by diverse SCF types of E3 ubiquitin ligases, such as beta-transducin repeat containing E3 ubiquitin protein (β-TRCP) [41] and F-box and WD repeat domain containing 7 (FBW7) [42], recently the similar modifications have also been observed for Cullin3^SPOP^ E3 ligase in recognizing its substrates. For example, SPOP could recognize CK1ε-phosphorylated SRC-3 degron [22], and CK1δ-phosphorylated ERG degron [24]. To figure out whether SPOP recognizes PDK1 in a phosphorylation dependent manner, we initially treated the lysates with λ protein phosphatase (λ-PPase) and found that the interaction of SPOP with PDK1 was dramatically reduced (Fig. 4A). Interestingly, a consensus motif (S/TxxxS/TxxxS/T) of GSK3β phosphorylation substrates was identified in the PDK1 degron region (Fig. 4B) [43], we sought to validate whether the GSK3β kinase could affect PDK1 stability by modulating PDK1/SPOP interaction. To this end, we performed in vitro kinase assays, and observed that GSK3β indeed directly phosphorylated PDK1 (Fig. S4A), while degron deletion of PDK1-Δ6S exhibited decreased GSK3β-mediated PDK1 phosphorylation (Fig. 4C). Consistently, active mutant form of GSK3β (GSK3β-S9A) could detectably decrease PDK1 protein levels in WT but not Δ6S PDK1 expressing cells (Fig. 4D). In keeping with these findings, GSK3β-S9A markedly enhanced, whereas GSK3β specific inhibitor (GSK3-i) abrogated SPOP interaction with and ubiquitination of PDK1 (Fig. 4E, S4B). As a result, GSK-i could prolong the half-life of PDK1 (Fig. S4C-D).

Given that GSK3β prefers to phosphorylate the primed phosphorylated substrates [44], we sought to examine whether other kinases are responsible for the prime phosphorylation of PDK1. Since CK1 family kinases involved in SPOP substrate phosphorylation [22, 24], we sought to evaluate whether CK1 kinases involved in GSK3β mediated PDK1 phosphorylation and subsequent degradation (Fig. 4F). Considering ERG and PDK1 shared similar sequence with a stretch of Ser/Thr residues (Fig. 4F), the phosphorylation of PDK1 in this degron may enhance the interaction between SPOP and PDK1 by CK1-mediated phosphorylation. Of note, CK1-inhibitors could not only block GSK3β-S9A-mediated PDK1 phosphorylation in its PolyS domain (Fig. 4G), but also compromise GSK3β-S9A-mediated PDK1 degradation (Fig. 4H). Furthermore, we sought to determine whether PDK1 underwent phosphorylation by CK1, and found that CK1δ and CK1ε, but not other CK1 isoforms, could interact with PDK1 (Fig. S4F). Notably, CK1δ promoted PDK1 protein degradation in a dose-dependent manner (Fig. 4I, S4G-H), which could be antagonized by the treatment of CK1 inhibitors IC261 or D4467 (Fig. 4I). Consistent with these results, depletion of *CKIδ* resulted in the accumulation of PDK1, coupled with increased AKT phosphorylation (Fig. 4J).

To determine whether CK1δ could directly phosphorylate PDK1, in vitro kinase assays were performed and showed that CK1δ could directly phosphorylate the bacterially purified recombinant PDK1 (Fig. S4I). Consistently, CK1δ-mediated PDK1 phosphorylation could be diminished by CK1 inhibitors (Fig. 4K, S4J). We further observed that degron-deleted PDK1-Δ6S could not be phosphorylated by CK1δ anymore (Fig. 4L). In agreement with this notion, CK1δ failed to boost the degradation of PDK1-Δ6S (Fig. 4M), coupled with the dissociation of CK1δ and PDK1 (Fig. 4N). Meanwhile, CK1δ could robustly enhance the interaction of PDK1 with SPOP (Fig. 4O), coupled with elevated PDK1 ubiqutination (Fig. S4K). Additionally, the potential GSK3β and CK1 phosphorylation residues have been shown undergoing phosphorylating by a mass spectrometry approach (Fig. S4L). Hence, these findings together suggest that CK1δ is a prime kinase for GSK3β phosphorylating PDK1, and coordinates for SPOP-mediated PDK1 ubiquitination and degradation (Fig. 4P).

### Cancer patients associated *PDK1* mutations, either in proximal degron or in GSK3β/CK1 phosphorylation region, display oncogenic roles by evading SPOP recognition

**Fig. 5:**
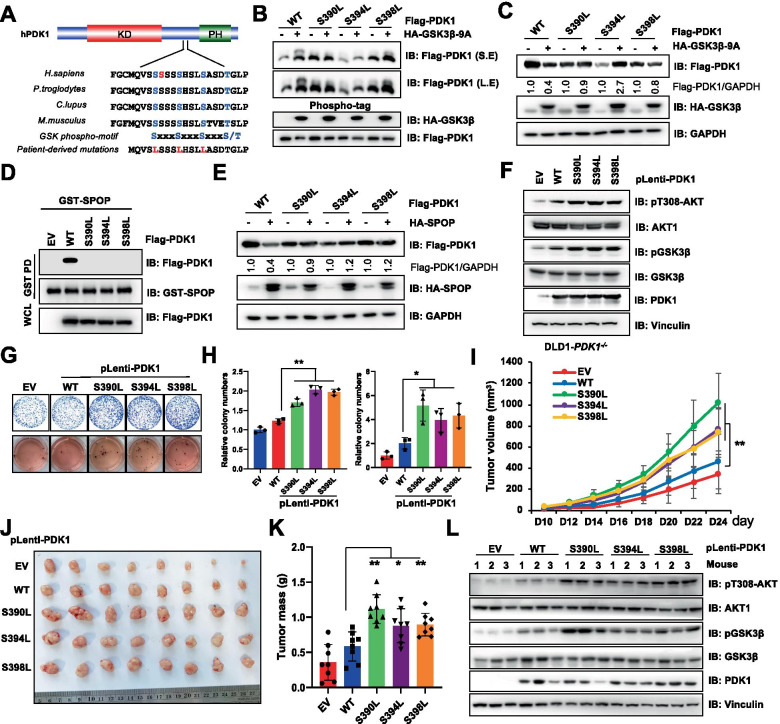
Patient-derived *PDK1* mutants block CK1/GSK3-mediated PDK1 phosphorylation and SPOP-mediated PDK1 degradation. **A** A schematic presentation of the patients associated *PDK1* mutations occurred around GSK3 mediated PDK1 phosphorylation region. **B** HEK293T cells transfected with indicated constructs were resolved by phospho-tag SDS-PAGE and immunoblotted with indicated antibodies. **C** IB analysis of WCL derived from HEK293T cells transfected with Flag-PDK1 (WT, S390L, S394L, S398L) and with/without HA-GSK3β-S9A. The relative protein levels of PDK1 were normalized with GAPDH. **D** IB analysis of WCL and GST-pulldown products derived from HEK293T cells transfected with indicated constructs and treated with MG132 (10 μM) for 10 h before harvested. **E** IB analysis of WCL derived from HEK293T cells transfected with Flag-PDK1 (WT, S390L, S394L, S398L) and with/without HA-SPOP. The relative protein levels of PDK1 were normalized with GAPDH. **F**–**H** IB analysis of DLD1-*PDK1* knockout cells stably infected with indicated constructs. Resulting cells were subjected to colony formation and soft agar assays (**G**), and the relative colony numbers were quantified (H). (mean ± SD, n = 3) (*t* test), *P < 0.05, **P < 0.01. **I-K** Cells generated in (**F**) were subjected to mouse xenograft assays. Tumor sizes were monitored (**I**), and dissected tumors were weighed (**J, K**). (mean ± SD, n = 8) (for I, and* t* test for K), *P < 0.05., **P < 0.01. **L** IB analysis of WCL derived from dissected tumor tissues

Although the genomic amplification of *PDK1* has been established in many cancers, the mutations, especially driver mutations for *PDK1* are not well evaluated. To this end, we analyzed the Cancer Genome Atlas (TCGA) and revealed that patients harbored *PDK1* mutations in proximal degron. In echoing the important roles of CK1 and GSK3β-mediated PDK1 phosphorylation and degradation, patients-associated mutations S390L, S394L and S398L were identified in cancer patients (Fig. 5A). Notably, these mutations could partially alleviate phosphorylation of PDK1 by GSK3β (Fig. 5B), resulting in resistance to GSK3β-mediated PDK1 degradation (Fig. 5C). Remarkably, *PDK1* mutations abrogated SPOP interaction with and ubiquitination of PDK1 (Fig. 5D, S4M-N), resulting in abolished SPOP-mediated PDK1 degradation (Fig. 5E). Functionally, re-introduction of these mutations into DLD1-*PDK1*^−/−^ cells could detectably increase pT308-AKT and promote colony formation, soft agar growth in cells (Fig. 5F-H), and tumor growth in xenografted mice (Fig. 5I-L, S4O). In conclusion, these findings indicate that GSK3β phosphorylation deficient mutations could confer PDK1 evasion from SPOP-mediated degradation, and contribute to increased AKT kinase activity and tumorigenesis.

**Fig. 6:**
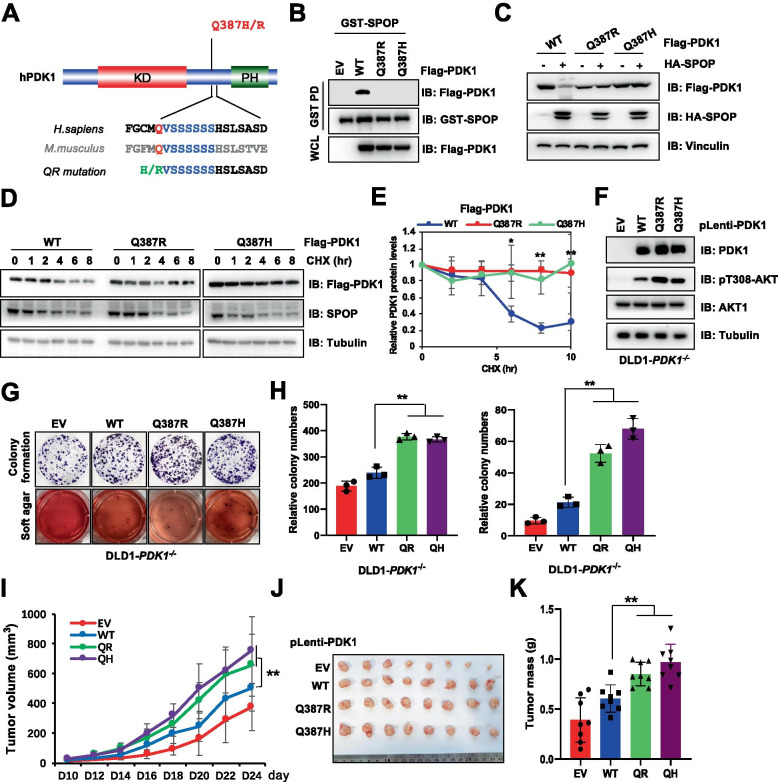
Patient-derived *PDK1* mutants adjacent degron block SPOP-mediated PDK1 degradation. **A** A schematic presentation of the patients-associated *PDK1* mutations. **B** IB analysis of WCL and GST-pulldown products derived from HEK293T cells transfected with indicated constructs. **C** IB analysis of WCL derived from HEK293T cells transfected with Flag-PDK1 (WT, Q387R, Q387H) and with/without HA-SPOP. **D**-**E** IB analysis WCL derived from of HEK293T cells transfected with indicated constructs. Where indicated, cells were treated with CHX (100 μg/ml) for the indicated time points before harvested. PDK1 protein abundance in (**D**) was normalized and quantified (**E**) (mean ± SD, n = 3) (*t* test), *P < 0.05. **F–H** IB analysis of DLD1-*PDK1* knockout cells stably infected with indicated constructs (**F**). Resulting cells were subjected to colony formation and soft agar assays (**G**), and the relative colony numbers were quantified (**H**). (mean ± SD, n = 3) (*t* test), **P < 0.01. **I-L** Cells generated in (**F**) were subjected to mouse xenograft assays. Tumor sizes were monitored (**I**), and dissected tumors were weighed (**J**, **K**) and subjected for IB analysis (**L**). (mean ± SD, n = 8) (ANOVA test for I, and *t* test for **K**), *P < 0.05., **P < 0.01

**Fig. 7:**
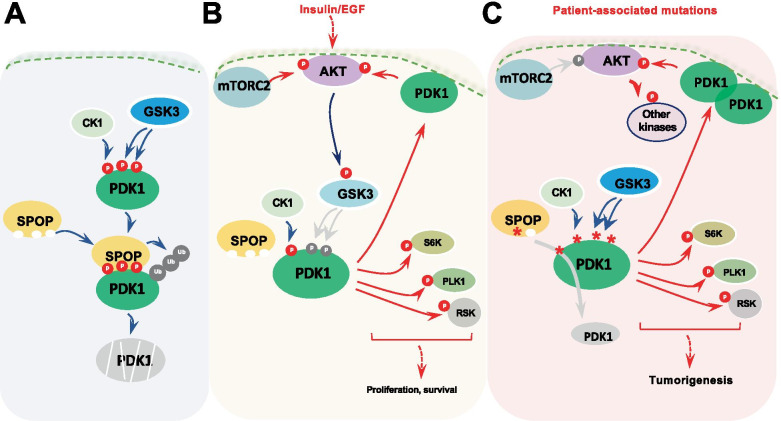
Proposed model of SPOP promotes the degradation of PDK1 in a CK1/GSK3-mediated phosphorylation dependent manner

Moreover, we also identified additional mutations, Q387H and Q387R, in PDK1 proximal degron region (Fig. 6A). As a result, both Q387H and Q387R mutations blocked the interaction between PDK1 and SPOP (Fig. 6B, S5A), impaired SPOP-mediated PDK1 degradation (Fig. 6C), prolonged PDK1 half-life (Fig. 6D-E) and decreased PDK1 ubiquitination (Fig. S5B). Compared with the positive charge basic property of histidine (H) and arginine (R), glutamine (Q) is a polar amino acid, which might be the reason for evading SPOP recognition. To further test this hypothesis, we mutated Q387 to A/L (nonpolar amino acids) or D/E (acidic amino acids), and observed that these mutations could not disturb the interaction between SPOP and PDK1 (Fig. S5C). Consistently, Q387R, but not other mutations could dramatically block SPOP-mediated PDK1 degradation (Fig. S5D). Functionally, re-introduction of Q387R or Q387H-PDK1 into DLD1-*PDK1*^−/−^ cells could significantly enhance AKT-pT308 (Fig. 6F), cellular colony formation and soft agar growth in cells (Fig. 6G-H, S5E), and tumor growth in vivo (Fig. 6I-K, S5F-G). Importantly, Q387H/R mutations could also enhance DLD1-*PDK1*^*−/−*^ cells chemo-therapeutic drug resistance compared with WT-PDK1 expressing cells (Fig. S5H-I). These findings indicate that patient derived *PDK1* mutations could markedly stabilize PDK1 protein and contribute to tumorigenesis by blocking SPOP-mediated decay of PDK1 (Fig. 7).

## Discussion

The PI3K-AKT pathway plays a pivotal role in a plethora of cancers including breast and prostate cancers [45, 46]. Of note, genetic mouse models have implied that *Pten* deletion is predominant to induce prostate neoplastic onset, especially after crossed with *Pml*^*−/−*^ or *P53*^*−/−*^ mice, indicating a pivotal role of AKT activation in prostate tumorigenesis. Although *PTEN* deletion only occurs around 10% in prostate cancer, whether and how aberrant AKT activation is promoted in *PTEN* intact prostate cancer is not well defined. As a tumor suppressor, *SPOP* mutations occur around 15% in prostate cancer, and display a mutual exclusion with *PTEN* deletion/mutations (Fig. S1M), indicating the potential connection of *SPOP* status and the PI3K-AKT signaling pathway. As the evidence from genetic mouse models has implied, conditional knockout of *Spop* in prostate readily promotes prostate tumorigenesis possibly due to largely activating the AKT kinase [27]. However, the underlying mechanism remains to be established. In this study, we demonstrated that SPOP directly ubiquitinated PDK1, the upstream kinase of AKT, subsequently promoting PDK1 degradation and retarding AKT kinase activity. This presents a potential mechanism for the physiological regulation of PDK1 turnover by E3 ligase SPOP, and further dictate the pathological conditions of prostate cancer, where patients conceived *SPOP* mutations, or *PDK1* degron-associated mutations facilitate tumorigenesis by activating AKT kinase.

Recently, emerging evidence shows that multiple proteins have been identified as the *bona fide* substrates of SPOP, especially the oncogenes AR, c-MYC, ERG and BRDs, all playing important roles in prostate tumorigenesis [17, 21, 25]. In our studies, we identified PDK1 as a novel substrate of SPOP, and provided the evidence that SPOP ubiquitinates and degrades PDK1 to repress AKT kinase. Biologically, knockdown *PDK1* could significantly decrease mutated *SPOP*-induced cancer cell malignant phenotypes. Given that the *Pdk1* knockout mice were previously reported to be lethal [47], to further evaluate the potential role of PDK1 in SPOP functions in vivo, the PDK1 lower expressing mutant *Pdk1*_*K465E*_ knock-in [48] or *Akt1* knockout mice [49] will be employed to cross with the *Spop* mutation knock-in mice [27], and provide robust evidence for the pathological roles of the PDK1-AKT pathway for SPOP tumor suppressor function. Conceivably, the PDK1-AKT pathway will possibly cooperate with other substrates of SPOP such as SRC, c-MYC or BRDs to promote the prostate tumorigenesis. However, the potential roles of SRC and BRDs in modulating AKT kinase activation are previously established [27, 28], indicating a more complicated regulation of SPOP on AKT kinase.

Interestingly, one study recently demonstrates that *SPOP* knockdown leads to PTEN accumulation and AKT inactivation in the clear cell renal carcinoma (ccRCC) cells [29]. Since SPOP has been shown to play an oncogenic role in targeting several tumor suppressors such as PTEN and ubiquitin specific peptidase 9 (USP9) under hypoxic conditions or *VHL* mutant ccRCCs [29, 50], here we demonstrate that under normal conditions or prostate cancer settings, SPOP could directly bind to and degrade PDK1 to repress AKT kinase activity. Thus, our studies suggest that SPOP would like to play dual functions to modulate AKT kinase activity either by targeting PTEN to activate AKT in ccRCCs or by targeting PDK1 to repress AKT in prostate cancer. The detailed mechanism for SPOP regulating AKT kinase possibly due to the potential modification or complex formation of SPOP in different tissues or hypoxic conditions worth further characterization.

Although PDK1 is reported as an auto-phosphorylation and activation kinase with less study for its regulation, here we found that both CK1 and GSK3β kinase directly phosphorylate PDK1. Interestingly, the phosphorylation occurred mainly in the PDK1 PloyS degron region, which dramatically blocked SPOP recognition and subsequent degradation of PDK1. Furthermore, GSK3β, the first established AKT substrate, has been previously revealed to negatively control AKT kinase activity by phosphorylating different substrates such as insulin receptor substrate (IRS1), tuberous sclerosis complex (TSC) [51]. Here we reported that GSK3β could directly phosphorylate AKT upstream kinase PDK1 to reduce PDK1 abundance and attenuate AKT kinase activity. In addition, as a prime kinase, CK1 has been shown to phosphorylate PDK1 initially, and then mediated GSK3β-induced PDK1 phosphorylation, after which SPOP bound PDK1 leading to its proteasome degradation (Fig. 7). However, due to multiple serine and threonine residing within/around PDK1-degron region, and several serine/threonine residues have been identified to be phosphorylated (Fig. S4L), it is kind of hard to distinguish the exact CK1 and GSK3β phosphorylation residues by the mass spectrometry approach.

## Conclusion

In this study we not only reveal the tumor suppressive role of SPOP by targeting PDK1 for degradation and AKT inactivation, but also highlight combating PDK1-AKT pathway as a potent strategy to treat *SPOP* mutant-driven prostate cancer (Fig. 7).

## Supplementary Information

**Additional file 1: Figure S1**. Identification of SPOP as a bona fide E3 ligase for PDK1. **Figure S2**. Cytoplasmic SPOP could not bind and degrade PDK1. **Figure S3**. SPOP degrades PDK1 by binding its degron. **Figure S4**. GSK3-mediated PDK1 phosphorylation promotes SPOP degrading PDK1. **Figure S5**. Patient-derived PDK1 mutants block SPOP-mediated PDK1 degradation.

## Data Availability

All data generated or analyzed during this study are included in this published article (and its supplementary information files, including 5 figures).

## Abbreviations

SPOP: Speckle-type POZ protein
PDK1: 3-Phosphoinositide-dependent protein kinase-1
AGC: Protein kinase A, G, and C family
AKT: Protein kinase B
S6K: Ribosomal protein S6 kinase beta-1
CRISPR: Clustered regularly interspaced short palindromic repeats
IF: Immunofluorescence
CK1: Casein kinase 1
GSK3: Glycogen synthase kinase 3
PI3K: Phosphoinositide 3-kinases
PTEN: Phosphatase and tensin homolog
NF1: Neurofibromatosis type 1
VHL: Von Hippel-Lindau
PP2A: Protein phosphatase 2A
KRAS: Ki-ras2 kirsten rat sarcoma viral oncogene homolog
mTORC2: Mammalian target of rapamycin complex 2
SGK: Serum- and glucocorticoid-inducible protein kinase
PLK1: Polo-like kinase 1
RSK: Ribosomal S6 kinase
PKC: Protein kinase C
PrCa: Prostate cancer
AR: Androgen receptor
CRPC: Castration-resistant prostate cancer
SRC-3: Steroid receptor coactivator 3
DEK: DEK proto-oncogene
Trim24: Tripartite motif containing 24
BRD4: Bromodomain-containing protein 4
ERG: ETS related gene 1
c-MYC: Proto-oncogene c-Myc
HEK293: Human embryonic kidney 293
FBS: Fetal bovine serum
MEFs: Mouse embryonic fibroblasts
CHX: Cycloheximide
EGFP: Green fluorescent protein
DNA: Deoxyribonucleic acid
shRNA: Small hairpin RNA
sgRNA: Single guide RNA
PCR: Polymerase chain reaction
IP: Immunoprecipitation
PD: Pull-down
WCL: Whole cell lysates
SDS-PAGE: Sodium dodecyl sulfate polyacrylamide gel electrophoresis
MG132: Carbobenzoxy-Leu-Leu-leucinal
ATP: Adenosine triphosphate
IPTG: Isopropyl β-d-1-thiogalactopyranoside
BSA: Bovine serum albumin
TBS: Tris-buffered saline
HIF1: Hypoxia-inducible factor 1
PBS: Phosphate-buffered saline
MS: Mass spectrometry
DTT: Dithiothreitol
HPLC: High performance liquid chromatography
FDR: False discovery rate
SD: Standard deviation
MDM2: Mouse double minute 2 homolog
RNF: Ring finger protein
BTB: Bric-a-brac
PIP_3_: Phosphatidylinositol (3,4,5)-trisphosphate
MATH: Meprin and TRAF homology
WT: Wild type
Φ: Nonpolar amino acid
Π: Polar amino acid
β-TRCP: Beta-transducin repeat containing E3 ubiquitin protein
FBW7: F-box and WD repeat domain containing 7
λ-PPase: λ Protein phosphatase
Pml: Progressive multifocal leukoencephalopathy
ccRCC: Clear cell renal carcinoma
USP9: Ubiquitin specific peptidase 9
IRS1: Insulin receptor substrate 1
TSC: Tuberous sclerosis complex
IB: Immunoblot
