# Supplementary material for: SPOP-mediated ubiquitination and degradation of PDK1 suppresses AKT kinase activity and oncogenic functions
Source: Mol Cancer. 2021 Aug 5;20:100. doi: 10.1186/s12943-021-01397-5 (PMC8340461; doi:10.1186/s12943-021-01397-5)
Supplement: Supplementary file 1 — Additional file 1: Figure S1. Identification of SPOP as a bona fide E3 ligase for PDK1. Figure S2. Cytoplasmic SPOP could not bind and degrade PDK1. Figure S3. SPOP degrades PDK1 by binding its degron. Figure S4. GSK3-mediated PDK1 phosphorylation promotes SPOP degrading PDK1. Figure S5. Patient-derived PDK1 mutants block SPOP-mediated PDK1 degradation. [file 12943_2021_1397_MOESM1_ESM.pdf]

Supplementary information

SPOP-mediated ubiquitination and degradation of PDK1 suppresses AKT kinase activity and oncogenic functions

Qiwei Jiang, Nana Zheng, Lang Bu, Xiaomei Zhang, Xiaoling Zhang, Yuanzhong Wu, Yaqing Su, Lei Wang, Xiaomin Zhang, Shancheng Ren, Xiangpeng Dai, Depei Wu, Wei Xie, Wenyi Wei, Yasheng Zhu, Jianping Guo

Including 5 figures

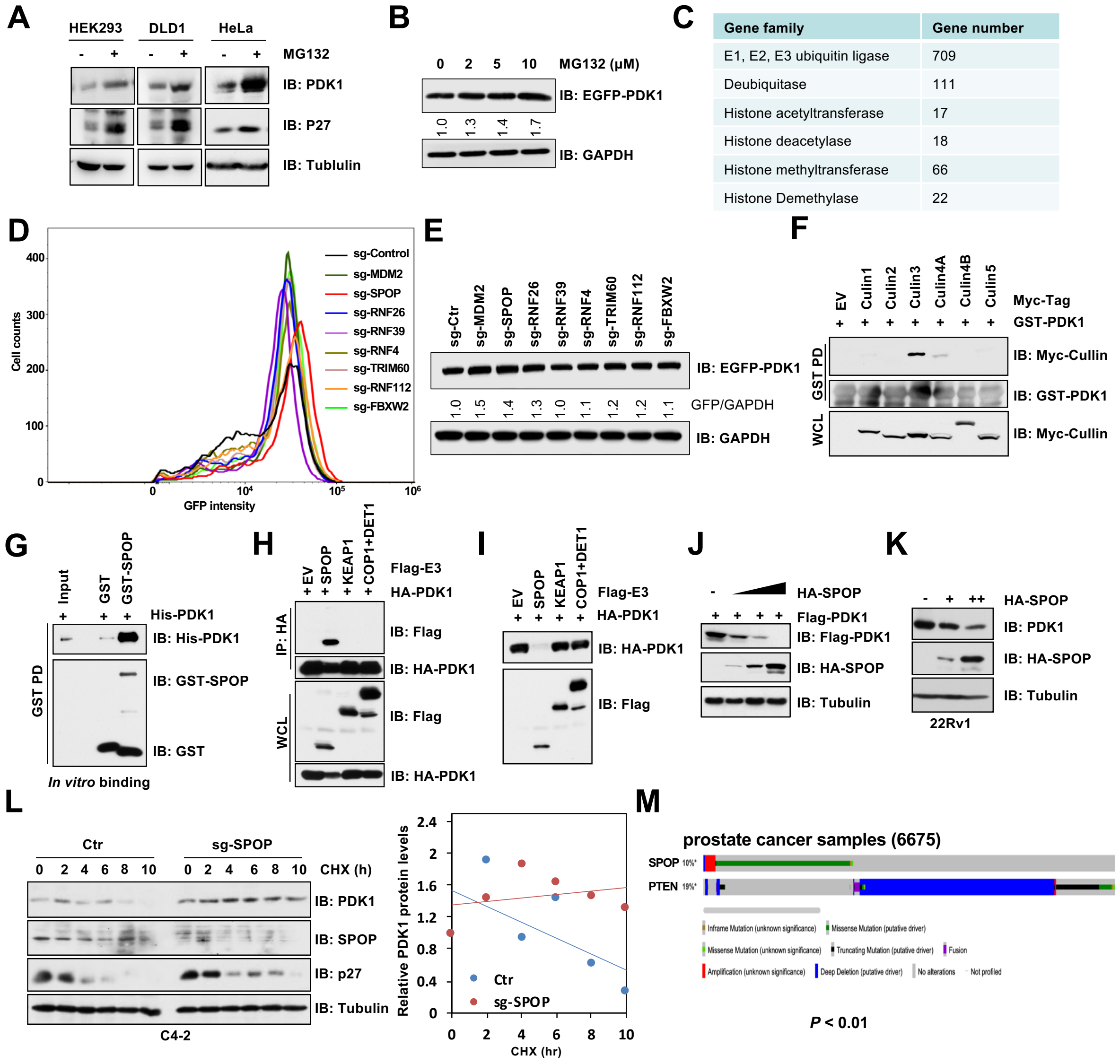

Gene family	Gene number
E1, E2, E3 ubiquitin ligase	709
Deubiquitase	111
Histone acetyltransferase	17
Histone deacetylase	18
Histone methyltransferase	66
Histone Demethylase	22

Figure S1. Identification of SPOP as a *bona fide* E3 ligase for PDK1. (A) IB analysis of WCL derived from different cells. Where indicated, cells were treated with MG132 (10 μM) for 10 hours before harvested. (B) HEK293T cells were infected with plenti-hygro-CMV-DsRed-IRES-EGFP-PDK1 constructs, and were selected with hygromycin (200 μg/ml) for 5 days to eliminate uninfected cells. Resulting cells were subjected for IB analysis after treated with different doses of MG132 for 8 hours before harvested. (C) A pooled genome-wide human sgRNA library targeting 943 annotated protein-coding genes with 11316 unique guide sequences (12 sgRNAs per gene). (D-E) Stable expression of DsRed/EGFP-PDK1 HEK293T cells were infected with lenti-CRISPR control or 8 genes of top candidate sgRNAs, then analyzed by flow cytometry (D) or IB (E). (F) IB analysis of WCL and GST-pulldown products derived from HEK293T cells transfected with GST-PDK1 and Myc-tagged Cullin family proteins. (G) GST pull-down analysis to show that bacterially purified recombinant His-PDK1 interacts with SPOP derived from HEK293T cells. (H) IB analysis of WCL and IP products derived from HEK293T cells transfected with indicated constructs and treated with MG132. (I) IB analysis of WCL derived from HEK293T cells transfected with HA-PDK1 and Flag tagged E3 proteins. (J) IB analysis of WCL derived from HEK293T cells transfected with Flag-PDK1 and different doses of HA-SPOP constructs. (K) IB analysis of WCL derived from 22Rv1 cells treated with increasing HA-SPOP. (L) IB analysis

of control and *SPOP* knockout C4-2 cells. Where indicated cells were treated with CHX (100 μg/ml) for the indicated time points before harvested. PDK1 protein abundance was quantified. (M) Data mining from TCGA database (total 6675 prostate cancer samples) demonstrates that *SPOP* mutations and *PTEN* mutations/deletion display mutually exclusive in prostate cancer.

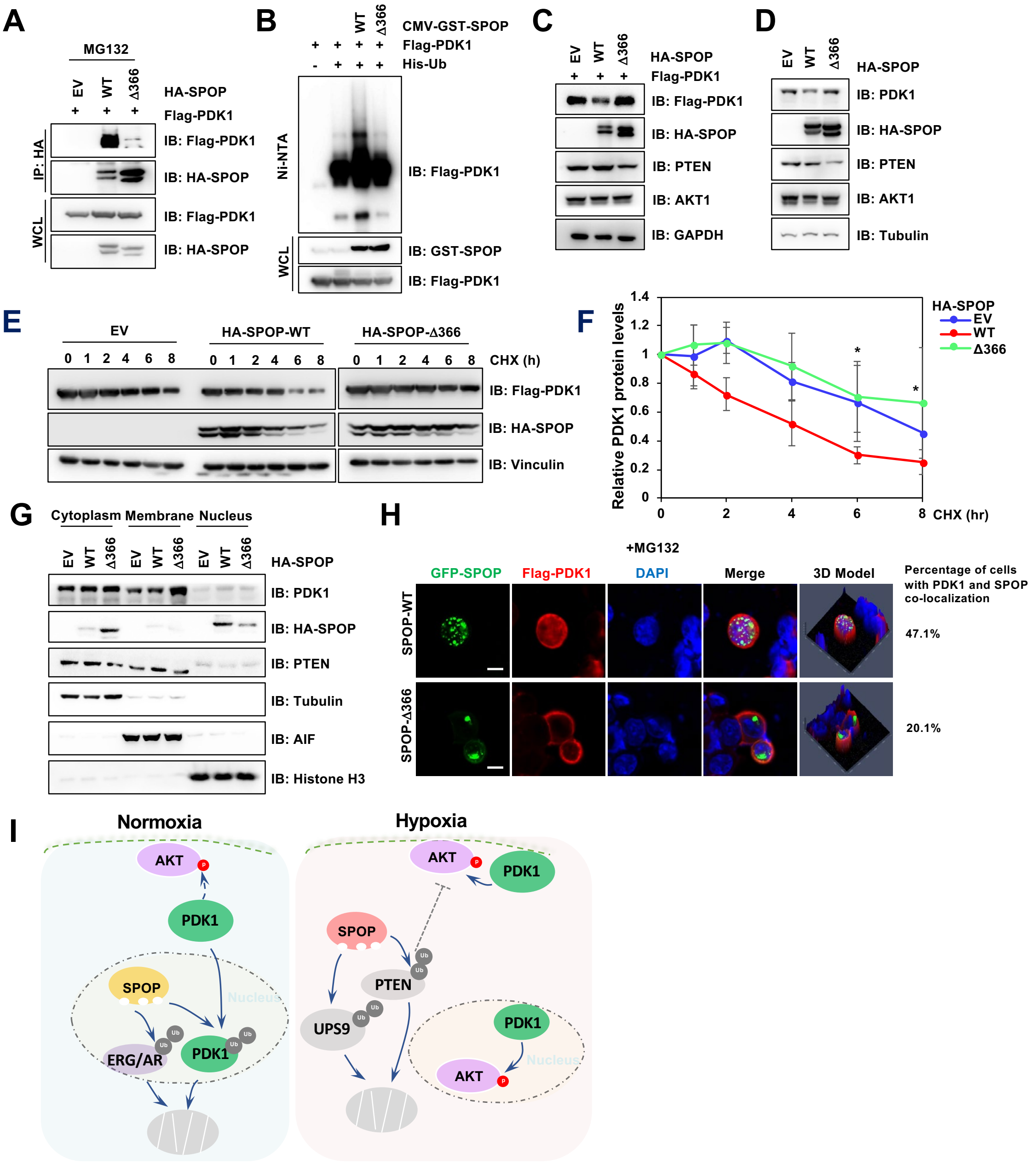

Figure S2. Cytoplasmic SPOP could not bind and degrade PDK1. (A) IB analysis of WCL and IP products derived from HEK293T cells transfected with indicated constructs. Where indicated, cells were treated with MG132 (10 μM) for 10 hours before harvested. (B) IB analysis of WCL and His pulldown products derived from HEK293T cells transfected with indicated constructs. Cells were treated with MG132 (10 μM) for 12 hours before harvested. (C-D) IB analysis of WCL derived from HEK293T cells transfected with HA-SPOP (EV, WT, Δ366) with (C) or without Flag-PDK1 (D). (E-F) IB analysis WCL derived from of HEK293T cells transfected with HA-SPOP (EV, WT, Δ 366). Where indicated, cells were treated with CHX (100 μg/ml) for the indicated time points before harvested. PDK1 protein abundance in (E) was normalized and quantified (F) (mean ± SD, n = 3) (*t* test), **P* < 0.05. (G) IB analysis of cell fractionations separated from HEK293T cells transfected with indicated constructs after treated with MG132 (10 μM) overnight. (H) Graphic representation of immunofluorescence staining of HEK293T cells

transfected with indicated constructs and treated with MG132 (10 μM) overnight. Scale: 10 μm; The relative quantified PDK1 and SPOP co-localization cell numbers have been labelled in right. (I) A schematic represents the different regulation of SPOP on the AKT kinase activity. On one hand, SPOP could directly target PDK1 for degradation to repress AKT activity in nucleus (Left panel); on the other hand, SPOP could transfer into cytoplasm to bind and degrade PTEN to activate the AKT kinase under hypoxic conditions (Right panel).

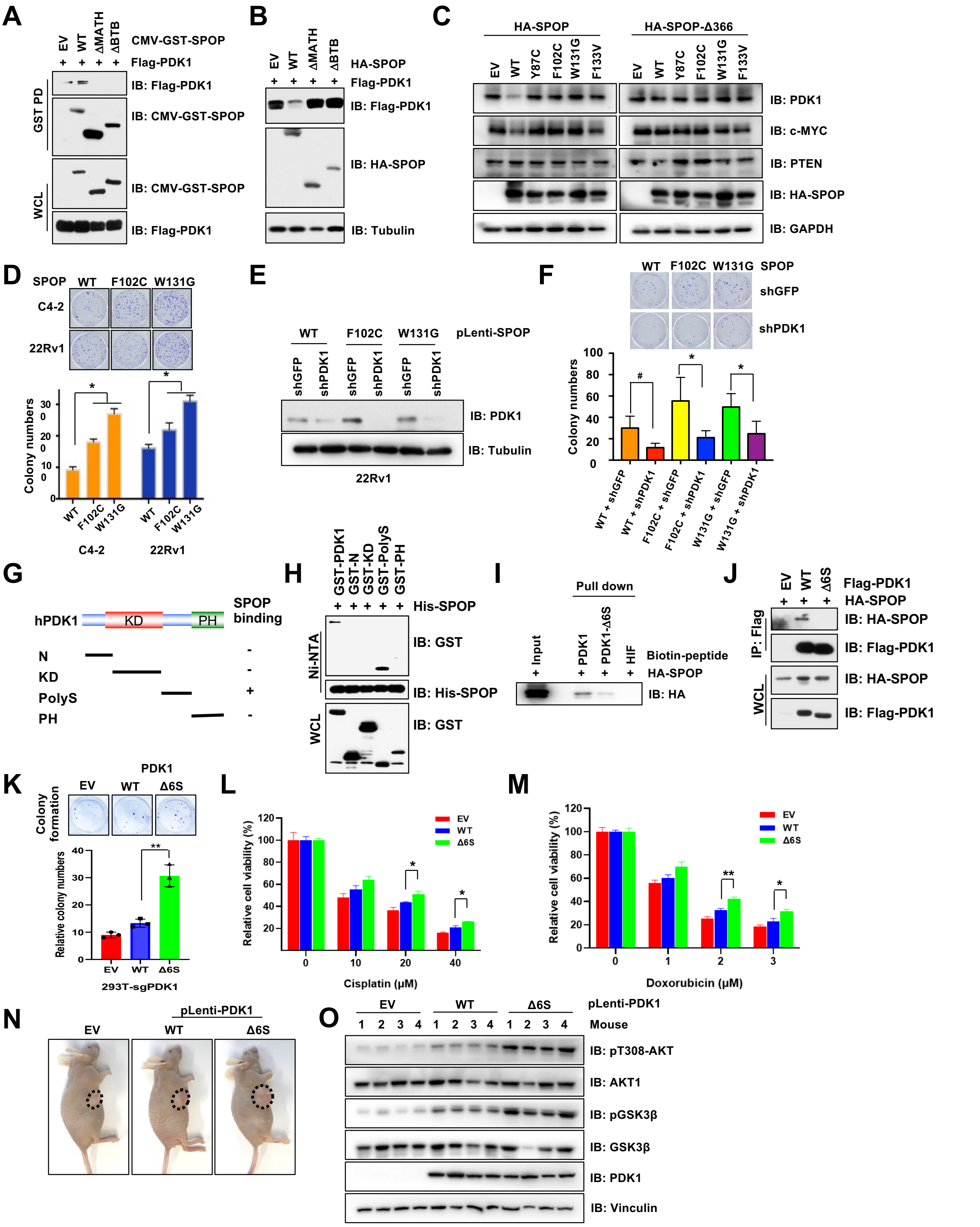

Figure S3. SPOP degrades PDK1 by binding its degron. (A-C) IB analysis of WCL and GST-pulldown products derived from HEK293T cells transfected with indicated constructs. (D) C4-2 and 22RV1 cell lines stably expressing WT or prostate cancer associated mutants *SPOP* were subjected to colony formation assays (top panel). Relative colony numbers were quantified (bottom panel). (mean ± SD, n = 3) (*t* test). $*P < 0.05$. (E-F) 22Rv1 cell lines stably expressed prostate cancer associated *SPOP* mutants were infected with shGFP or shPDK1 lentivirus. Cells

were selected with puromycin (1 μg/ml) for 72 hours to eliminate uninfected cells and used for IB analyses (E). Resulting cells were subjected to colony formation assays (F, top panel), quantified and plotted (F, bottom panel) (mean ± SD, n = 3) (t test). $^{\#}P > 0.05$, $^{*}P < 0.05$. (G-H) PDK1 structure was overviewed (G), and constructed into GST-fusion proteins. These PDK1 fragments were expressed in HEK293T cells and subjected to His-pulldown assays with bacterially purified His-SPOP protein (H). (I) Indicated peptides were incubated with WCL derived from HEK293T cells transfected with HA-SPOP, and precipitated with streptavidin. (J) IB analysis of WCL and IP products derived from HEK293T cells transfected with indicated constructs. (K) HEK293T-*PDK1* knockout cells stably infected with indicated constructs were subjected to colony formation assays (top panel). The relative colony numbers were quantified (bottom panel). (mean ± SD, n = 3) (*t* test). $^{**}P < 0.01$. (L-M) DLD1-*PDK1* knockout cells stably infected with indicated constructs were cultured in 10% FBS-containing medium with the indicated concentrations of Doxorubicin or Cisplatin for 48 hours and then subjected for the cell viability assays. Relative cell viabilities were quantified (J). (mean ± SD, n = 3) (*t* test). $^{*}P < 0.05$; $^{**}P < 0.01$. (N) Cells generated in (Fig. 3G) were subjected to mouse xenograft assays. n = 8 mice. (O) IB analysis of WCL derived from dissected tumor tissues.

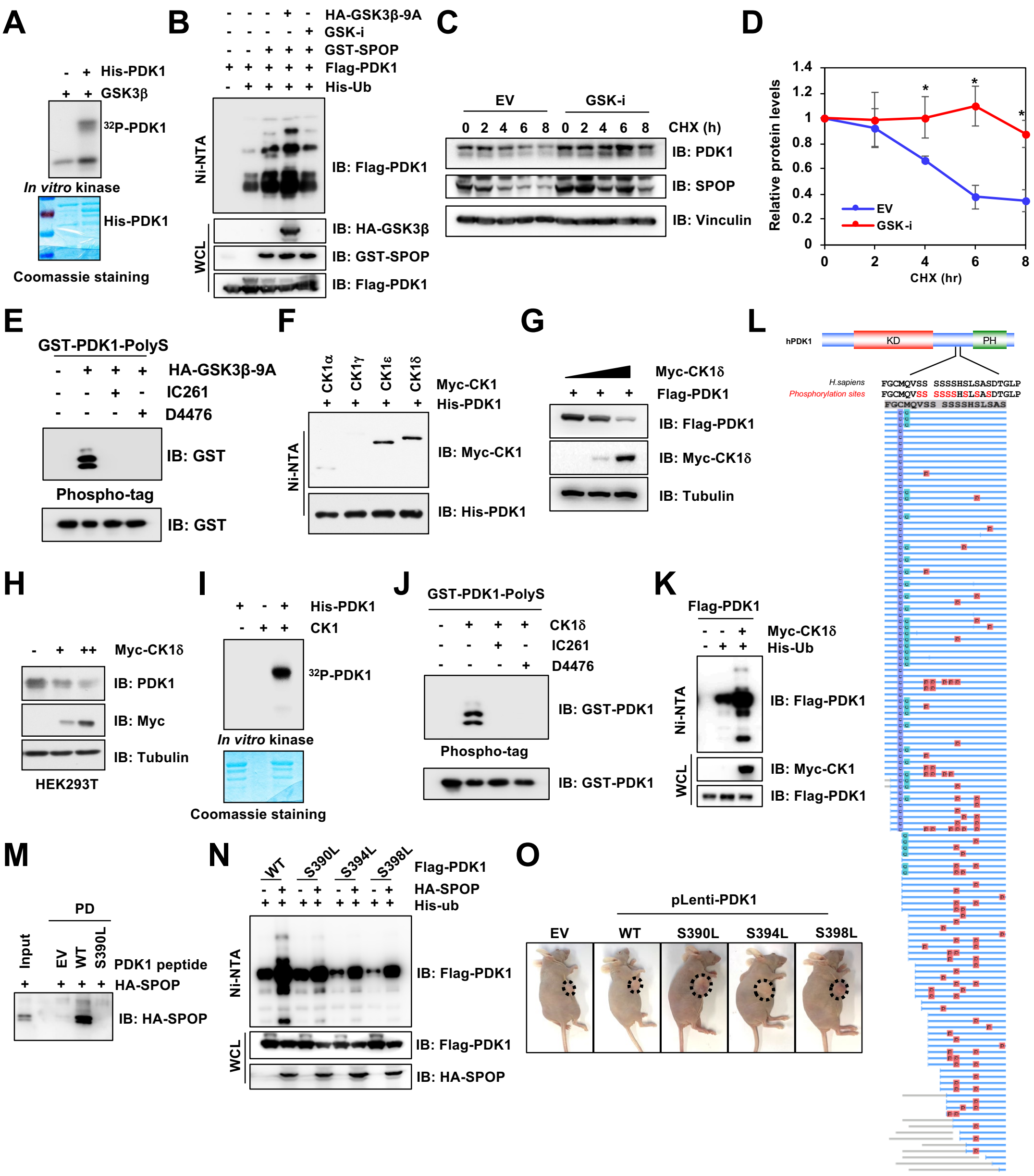

Figure S4. GSK3-mediated PDK1 phosphorylation promotes SPOP degrading PDK1. (A) *In vitro* kinase assay was performed with bacterially purified PDK1 as substrate, and recombinant GSK3β protein as the kinase source. ^{32}P isotope-ATP was used for autoradiography of phosphorylated PDK1. (B) IB analysis of WCL and His pulldown products derived from HEK293T cells transfected with indicated constructs. Where indicated, cells were treated with GSK3β inhibitor CHIR-99021 (10 μM) for 10 hrs before harvested. (C-D) IB analysis of WCL derived from indicated cells treated with/without GSK3β inhibitor CHIR-99021 (10 μM) overnight. Where indicated, cells were treated with CHX (100 μg/ml) for the indicated time points before harvested (C). PDK1 protein abundance was normalized and quantified (D) (mean ± SD, n = 3) (*t* test), **P* < 0.05. (E) HEK293T cells transfected with indicated constructs were resolved by phospho-tag SDS-PAGE, and immunoblotted with indicated antibodies. Where indicated, cells were treated with CK1 inhibitor IC261 (50 μM) or D4476 (20 μM) for 10 hrs before harvested. (F) IB analysis of WCL and His pulldown products derived from HEK293T cells transfected with

indicated constructs. (G-H) IB analysis of WCL derived from HEK293T cells transfected with indicated constructs. (I) *In vitro* kinase assay was performed with bacterially purified PDK1 as substrate, and recombinant CK1 protein as the kinase source. ^{32}P isotope-ATP was used for autoradiography of phosphorylated PDK1. (J) HEK293T cells transfected with indicated constructs were resolved by phospho-tag SDS-PAGE, and immunoblotted with indicated antibodies. Where indicated, cells were treated with CK1 inhibitor IC261 (50 μM) or D4476 (20 μM) for 10 hours before harvested. (K) IB analysis of WCL and His pulldown products derived from HEK293T cells transfected with indicated constructs. Cells were treated with MG132 (10 μM) for 10 hours before harvested. (L) Mass spectrometry analysis of PDK1 phosphorylation was performed with implicated Flag-PDK1 from HEK293T cells. The peptides with/without phosphorylation residues within the ployS domain were summarized. The serine/threonine residues with phosphorylation modification were labeled red. (M) Indicated peptides were incubated with WCL derived from HEK293T cells transfected with HA-SPOP, and precipitated with streptavidin. (N) IB analysis of WCL and His pulldown products derived from HEK293T cells transfected with indicated constructs. Cells were treated with MG132 (10 μM) for 10 hours before harvested. (O) Cells generated in (Fig. 5F) were subjected to mouse xenograft assays. n = 8 mice.

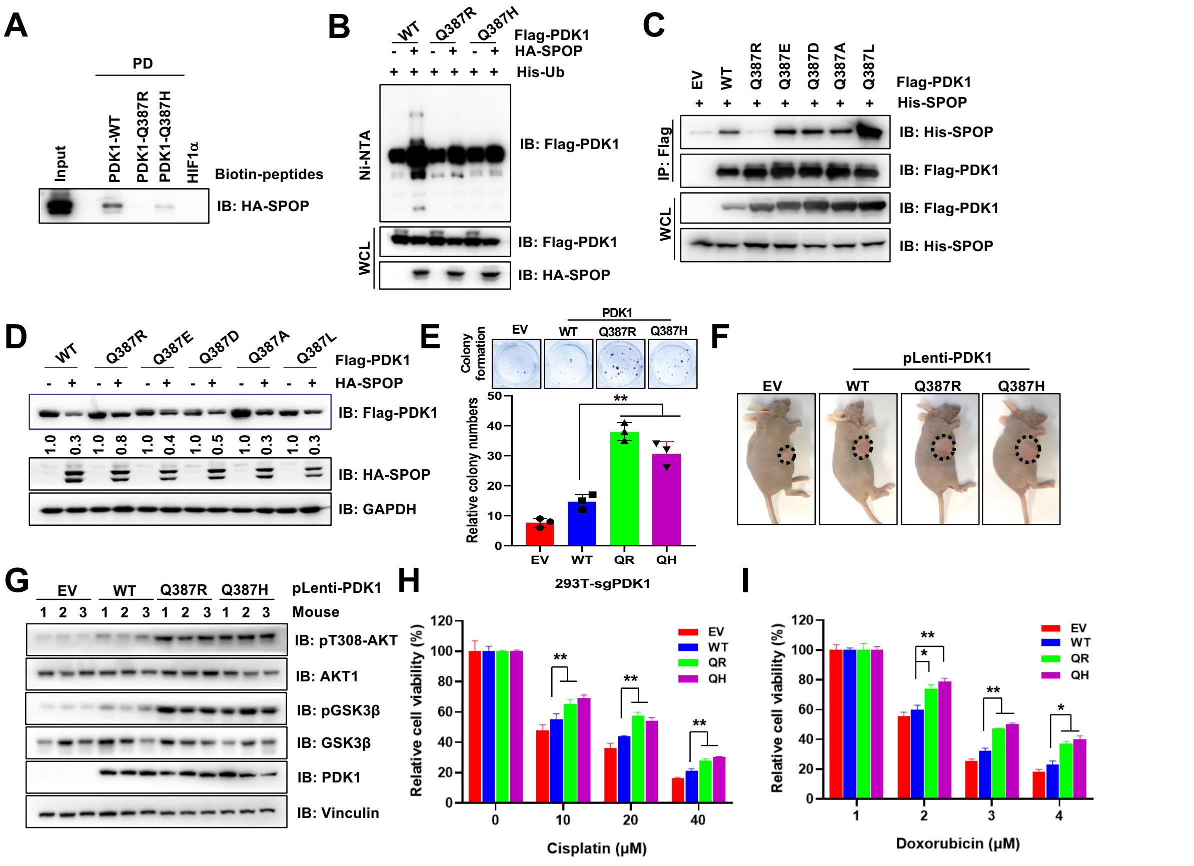

Figure S5. Patient-derived *PDK1* mutants block SPOP-mediated PDK1 degradation. (A) Indicated peptides were incubated with WCL derived from HEK293 cells transfected with HA-SPOP, and precipitated with streptavidin beads. (B) IB analysis of WCL and His pulldown products derived from HEK293T cells transfected with indicated constructs. Cells were treated with MG132 (10 μM) for 12 hours before harvested. (C-D) IB analysis of WCL and IP products derived from HEK293T cells transfected with indicated constructs. The relative protein levels of PDK1 were normalized with GAPDH. (E) HEK293T-*PDK1* knockout cells stably infected with indicated constructs were subjected to colony formation assays (top panel). The relative colony numbers were quantified (bottom panel). (mean ± SD, n = 3) (*t* test). $^{**}P < 0.01$. (F) Cells generated in (Fig. 6F) were subjected to mouse xenograft assays. n = 8 mice. (G) IB analysis of WCL derived from dissected tumor tissues. (H-I) DLD1-*PDK1* knockout cells stably infected with indicated constructs were cultured in 10% FBS-containing medium with the indicated concentrations of Cisplatin or Doxorubicin for 48 hours and subjected for the cell viability assays. The relative cell viabilities were quantified. (mean ± SD, n = 3) (*t* test), $^{*}P < 0.05$; $^{**}P < 0.01$.
